# Evaluation of the *OPTC *gene in primary open angle glaucoma: functional significance of a silent change

**DOI:** 10.1186/1471-2199-8-21

**Published:** 2007-03-14

**Authors:** Moulinath Acharya, Suddhasil Mookherjee, Ashima Bhattacharjee, Sanjay KD Thakur, Arun K Bandyopadhyay, Abhijit Sen, Subhabrata Chakrabarti, Kunal Ray

**Affiliations:** 1 Molecular & Human Genetics Division, Indian Institute of Chemical Biology, Kolkata, India; 2 Regional Institute of Ophthalmology, Medical College, Kolkata, India; 3 Dristi Pradip, Jodhpur Park, Kolkata, India; 4 LV Prasad Eye Institute, Hyderabad, India

## Abstract

**Background:**

We investigated the molecular basis of primary open-angle glaucoma (POAG) using Opticin (*OPTC*) as a candidate gene on the basis of its expression in the trabecular meshwork cells involved in the disease pathogenesis. Two hundred POAG patients and 100 controls were enrolled in this study. The coding sequence of *OPTC *was amplified by PCR from genomic DNA of POAG patients, followed by SSCP, DHPLC and DNA sequencing. Subsequent bioinformatic analysis, site-directed mutagenesis, quantitative RT-PCR and western blot experiments were performed to address the functional significance of a 'silent' change in the *OPTC *coding region while screening for mutations in POAG patients.

**Results:**

We detected two missense (p.Glu66Gly & p.Ile89Thr) and one silent change (p.Phe162Phe; c.602 C>T) that was present in 3 different patients but in none of the 100 controls screened. The mutant (c.602T) mRNA was predicted to have remarkably different secondary structure compared to the wild-type transcript by *in silico* approaches. Subsequent wet-lab experiments showed lower expression of the gene both at the mRNA and protein levels.

**Conclusion:**

Our study suggests *OPTC *as a candidate gene for POAG. Further, it highlights the importance of investigating the 'silent' variations for functional implication that might not be apparent from only *in silico* analysis.

## Background

Glaucoma comprises a group of neurodegenerative disorders of the eye characterized by a progressive loss of retinal ganglion cells and atrophy of the optic nerve [1]. With a multifactoral etiology, glaucoma account for 12.3% blindness worldwide [2]. Primary Open Angle Glaucoma (POAG) is the most common form of the disease [3] where the vision is lost in a progressive manner and, if untreated, results in bilateral blindness. About 33 million people are affected with POAG [3]. It usually gives no indication to the patient until there is appreciable and irreversible loss of the field of vision. Normal Tension Glaucoma (NTG), juvenile glaucoma, chronic open angle glaucoma are common subtypes of POAG. So far three different genes, *Myocilin *(*MYOC*) and *Optineurin *(*OPTN*) and *WDR36 *[4-6] and eleven additional chromosomal loci have been reported to be linked to POAG [5-14]. Also digenic form of the disease has been reported based on mutations in *MYOC *and *CYP1B1 *genes [15].

Recent studies have implicated genes that are expressed in the optic nerve head (*PAD2*) [16] and the trabecular meshwork (Cochlin) [17] to be involved in POAG pathogenesis. We examined *Opticin *(*OPTC*) as a candidate for POAG since it is expressed in the trabecular meshwork – the ocular site where the POAG related pathogenesis occurs [18]. *OPTC *encodes a protein of 332 amino acids and a member of class III small leucin-rich repeat protein (SLRP) family [19,20]. The gene is located on chromosome 1q31-q32 within an age-related macular degeneration (AMD) susceptibility locus [21,22] and examined for nucleotide variants in both AMD and POAG patients [18].

We, therefore, screened *OPTC *as candidate in 200 eastern Indian POAG patients to decipher the underlying complexity of the disease and attempted to broaden our understanding regarding its pathogenesis.

## Results

### Mutation and SNP profile in *OPTC*

**Figure 1:**
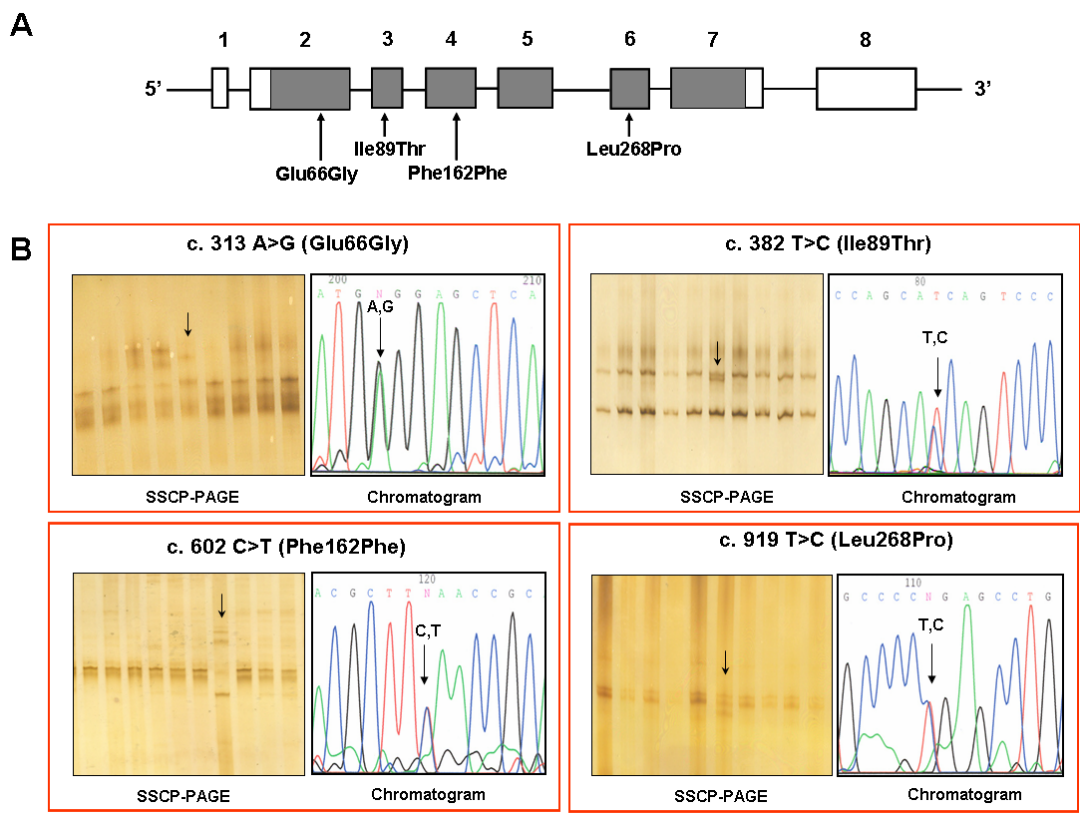
**Nucleotide variants in *OPTC***. (***A***): Gene structure of *Opticin*. Bars and lines represent the exons and introns, respectively. Exon numbers and mutation/polymorphism therein are indicated. Within exons shaded and open boxes show coding sequences and untranslated regions, respectively. (***B***): SSCP images along with chromatograms showing nucleotide changes in *OPTC *of POAG patients identified in Table 1.

**Figure 2:**
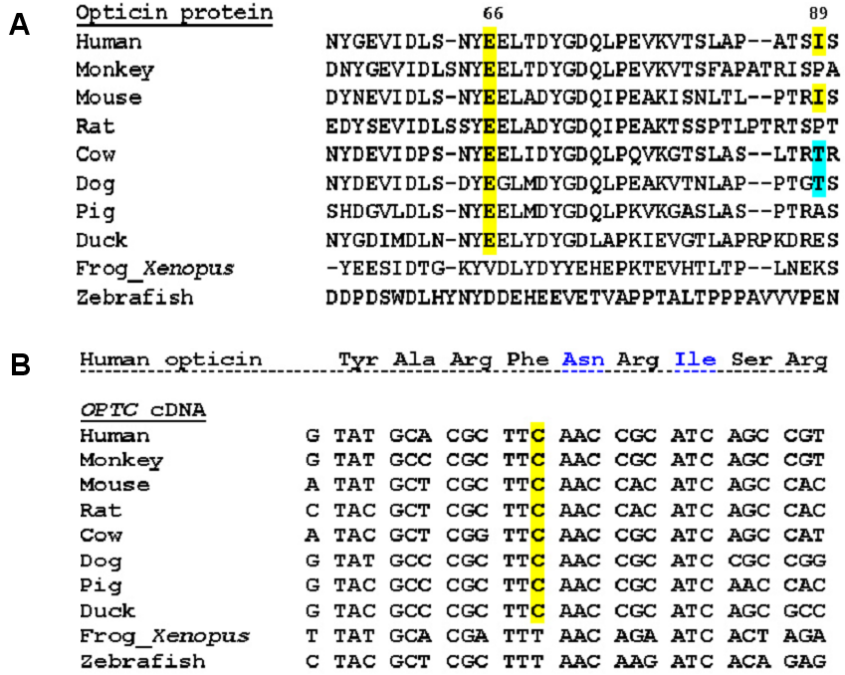
**Evolutionary conservation status of the silent change at cDNA level and other two nucleotide variants at protein level found in *OPTC***. (***A***): Multiple sequence alignment of opticin protein showing the conservation status of p.Glu66 (c.313A>G) residue and p.Ile89 (c.382T>C) residue across different vertebrate species. The sources of the sequences are NCBI: NP_055174.1 (human), XP_001102193.1 (monkey), NP_473417.1 (mouse), XP_222641.4 (rat), NP_991339.1 (cow), NP_001003056.1 (dog), NP_999173.1 (pig), NP_989804.1 (duck), NP_001016499.1 (*Xenopus*), ABB53599.1 (zebrafish). Except two lower vertebrates (*Xenopus *and zebrafish), p.66 (c.313A>G) is conserved in all higher vertebrate species, while p.Ile89 (c.382T>C) is conserved only in human and mouse. In both cases, multiple sequence alignment was done using ClustalW of EMBL-EBI [39] (***B***): Multiple sequence alignment of OPTC cDNA shows conservation of the wobble base for p.Phe162 which is altered (C>T) in case of a POAG patient (GL42 in Table 1). The sources of the sequences are GenBank: NM_014359 (human), XM_001102193.1 (monkey), NM_054076.1 (mouse), XM_001061460.1 (rat), NM_205770.1 (cow), NM_001003056.1 (dog), NM_214008.1 (pig), NM_204473.1 (duck), NM_001016499.2 (*Xenopus*) and NM_001003583.1 (zebrafish). Also, corresponding amino acid sequence of human opticin (NP_055174.1) is given above; except two lower vertebrates (*Xenopus *and zebrafish), c.602C (p.Phe162) is conserved in all higher vertebrate species.

A novel change (c.313A>G) that would result in a non-conservative substitution (p.Glu66Gly) was detected in exon 2 of *OPTC *(Fig 1B) in a sporadic patient in heterozygous condition (Table 1). The suspect allele was not detected in any of 100 control individuals screened for variation in the *OPTC*. In addition, the encoded amino acid residue (p.Glu66) being affected is conserved in higher vertebrates (i.e. human, mouse, dog, pig, cow & duck) among all the species for which the sequence of OPTC homologue is available (Fig 2A). Also, a SIFT score of 0.03 suggests functional relevance of the protein resulting from the variant allele (Table 1). The patient (36 years old male patient, GL 131 in Table 1) had a total loss of vision, with no PL (perception of light) in the right eye and with the ability to count fingers at one-foot distance with PR (projection of rays) in the left eye. When we first met the patient he had already undergone trabeculectomy in his right eye and before the surgery he almost lost the vision in the right eye. He had maximum IOP of 16 and 42 mm of Hg in the right & the left eye, respectively. There was no previous record for IOP in his right eye, and the value at post-operative stage was 16 mm of Hg. The cup to disc ratio could not be determined in the right eye due to presence of cataract and hazy media and 0.8 in the left eye. Gonioscopy revealed open angle (grade III – IV). This nucleotide change was not found in 100 healthy control samples (Table 1).

The second nucleotide alteration (c.382 T>C, p.Ile89Thr; Fig. 1B) was identified in heterozygous condition in exon 3 of OPTC gene of a 65 year old male sporadic patient (GL77 in Table 1) who was also heterozygous for a reported mutation (c.1960 G>A; Arg545Gln) in *OPTN *[23]. The IOP of this patient, recorded in aided condition, was 25 & 18 mm of Hg in the right and the left eye, respectively. The lower IOP value in the left eye was due to the medication used. Unlike the other variant (c.313A>G), the amino acid residue (p.Ile89) for the wild type allele (c.382 T) for this nucleotide change (c.382 T>C) is conserved only in human and mouse (Fig 2A), and gives a non-significant (0.66) SIFT score (Table 1). Though the nucleotide change was not identified in 100 control individuals, it appears unlikely to be pathogenic.

Both the nucleotide variants (c.313A>G, p.Glu66Gly & c.382 T>C, p.Ile89Thr) were examined for aberrant mRNA structure and splice variation, if any, using the *in silico* tools (RNAdraw, ESE finder and RESQUE ESE) but did not find any significant variation in predicted RNA structure or splice alteration. Patients carrying these two novel changes were sporadic in nature and their family members were not available to determine the disease status in the relatives and the segregation pattern of the variant alleles.

A reported SNP [18] (c.919 T>C; p.Leu268Pro) identified in a patient (Fig 1B) was scored in other patients and controls by Bpu10I RFLP assay. However, no significant difference was observed between the patients and control subjects either in allele frequencies (p = 0.247 under df = 1) or genotype distribution (p = 0.4136 under df = 2). The heterozygosity of the SNP in patient and control groups was found to be 0.25 and 0.19, respectively.

### Functional importance of a 'silent' change in *OPTC *coding region

A novel change (c.602 C>T) in exon 4 of *OPTC *(Fig 1B), that on conceptual translation would remain silent (p.Phe162Phe), was detected in heterozygous condition in a sporadic patient but none in 100 control individuals (Table 1). The patient, a 46 year old male patient (GL 42 in Table 1) with unilateral glaucoma in the right eye, presented with a visual acuity of 6/18 (unaided), IOP of 17 mm of Hg and a cup to disc ratio of 0.8. Gonioscopy revealed open angle (grade III – IV) and a mature and complicated cataract in the right eye. The visual field analysis in the right eye showed localized defect near fixation point in the infero-temporal quadrant and tubular vision. He was under anti-glaucoma medication in the right eye. The left eye was traumatic with a history of penetrating injury at ages of 12 and 14 years. There was no perception of light on projection in the left eye. Incidentally younger brother (GL 116) of the proband (GL42) is also heterozygous for the 'silent' change (c.602 C>T) who was a 'suspect' for POAG with raised IOP but no significant C:D ratio or visual field change. However, this individual later did not come for follow up and could not be traced despite best efforts.  No splice alteration was predicted to be caused by the variant allele as determined by *in silico* analysis using ESE finder and RESQUE ESE.

**Figure 3:**
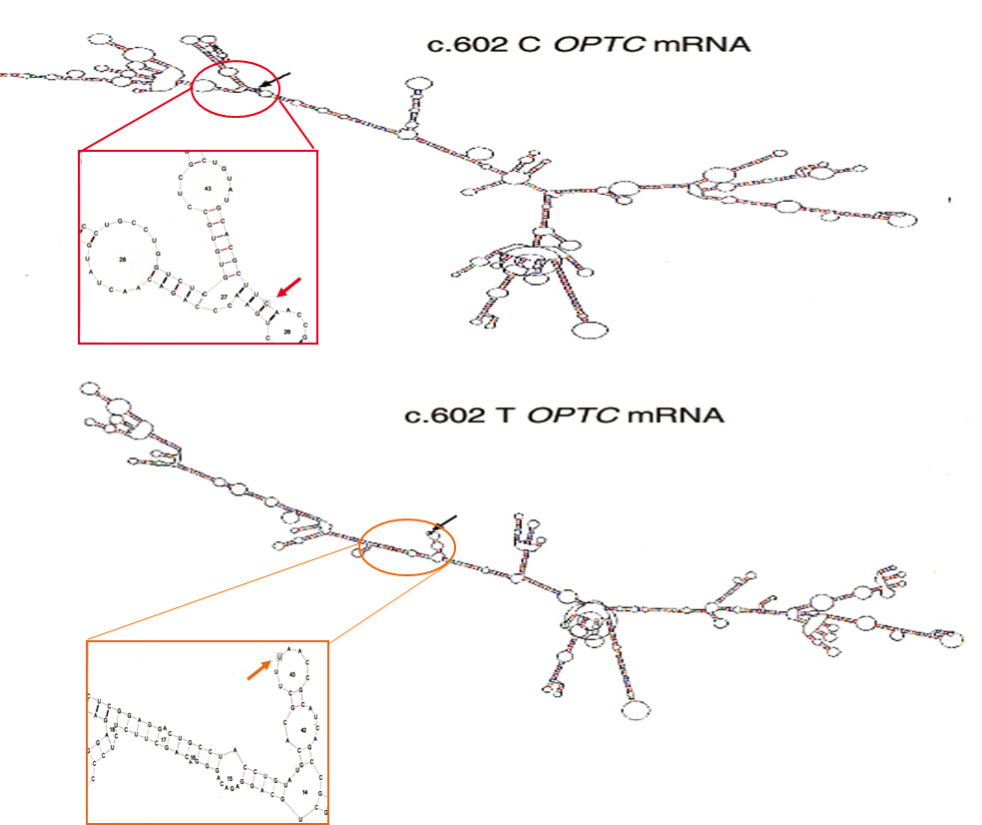
**Difference in the mRNA secondary structure between c.602C and c.602T variant in *OPTC***. Both the structures were predicted using the default parameter of RNAdraw (calculation temp. 37°C). Region of the mRNA harboring the variant nucleotide (indicated by arrow) has been enlarged for both the alleles to demonstrate the local structural alteration as predicted by RNAdraw.

**Figure 4:**
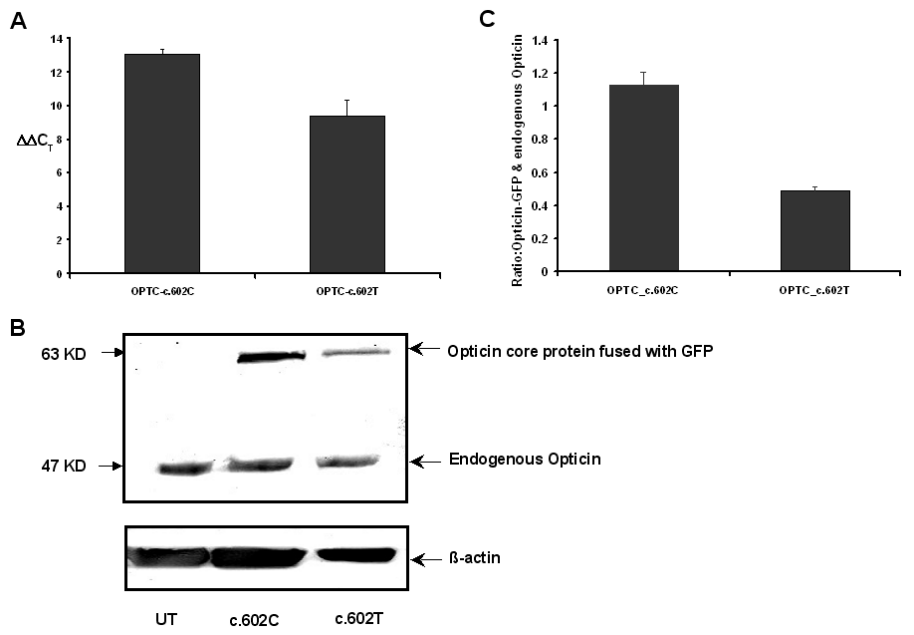
**Difference in mRNA and protein expression levels between c.602C and c.602T variants in *OPTC***. (***A***): Quantiative RT-PCR showing expression of c.602C and c.602T in mRNA level. The difference in mRNA expression is represented by ΔΔC_T _value. (***B***): Western blot of two variants of Opticin core protein fused with GFP as well as endogenous Opticin in RPE cells. UT represents the untransfected RPE cells. (***C***): Bar diagram showing the mean ± SD of densitometric scanning of western blots done thrice, that indicates much less expression of OPTC-c.602T variant (43%) than the normal one (OPTC-c.602C). Expression of transfected protein (Opticin-GFP) was normalized with endogenous Opticin.

Interestingly, though the variant nucleotide is the third base of the codon and prone to wobble, at mRNA level, it is evolutionarily conserved in all higher vertebrates among the species for which the sequence of OPTC homologue is available (Fig 2B). There are two codons (TTC & TTT) for Phe that, as per latest NCBI data have no significant difference in the relative usage in human proteins (i.e. 20 vs 17). This provoked us to look for the stability of this variant at mRNA level using RNAdraw. We observed that though the variation of structure energy (8 kJ) between variant (-1525.24kJ) and normal *OPTC *mRNA (-1533.11kJ) did not appear to be significant enough affecting stability of the variant, a large change in the secondary structure of mRNA was apparent (Fig 3). We reasoned that an altered structure of the mRNA, if any, might influence the efficiency of translation of the transcript due to a single nucleotide change (c.602 C>T). In the NCBI SNP database we found another silent change (c.518C>T, p.Leu134Leu, rs747814) 84 bp upstream but not in the close proximity of the silent change (c.602C>T) under investigation. The 5' silent SNP (c.518C>T) was tested for change in the mRNA secondary structures between the two alleles and no significant variation was observed. To find out its downstream implication, the variant (c.602T) was created by site directed mutagenesis in the *Opticin *cDNA cloned in pEGFPN1 and subsequently transfected both wild type (c.602C) and variant (c.602T) in the retinal pigment epithelium (RPE) cells. Transfection experiments were done in 3 to 4 times followed by subsequent quantitative RT-PCR (QRT) and western blot analysis. From the QRT data it was evident that the mRNA amount of the silent variant (c.602T) was lower (the difference in ΔΔC_T _value between c.602C and c.602T was found to be 3.73) with respect to wild type (c.602C) (Fig 4A). Further, western blot analysis using anti-opticin antibody clearly showed that expression of the mutant protein (c.602T) was lower (43%) compared to the native protein (c.602C) (Fig 4B &4C). Both the GFP fused proteins were normalized with the endogenous Opticin in RPE cells.

## Discussion

The complex nature of POAG warrants identification of genes that are expressed in the TM tissues, retinal ganglion cells and optic nerve head in order to understand the underlying molecular mechanisms leading to the disease pathogenesis. Recent studies in this direction have identified genes that could be potentially involved in the disease pathology [1]. Among these candidate genes, *Myocilin *mutations account for about 3–4% of open angle glaucoma (OAG) [4], with the Gln368Stop nonsense variant accounting for many, but not all, of the adult onset OAG cases [24,25]. Most of the other *MYOC *variants are missense mutations usually associated with a JOAG phenotype. A small number of mutations in *Optineurin *are found in families in which most affected individuals have NTG [23]. Mutations in *WDR36 *have been observed to be mostly associated with adult POAG patients with high levels of IOP [6]. Much remains to be understood about the underlying mechanisms by which mutations in these genes predispose to glaucoma and most cases cannot be accounted for by these three genes. In a previous study, *OPTC *was examined in 142 patients with ocular problem: 45 with age-related macular degeneration (AMD), 10 with AMD plus POAG or NTG, and 87 individuals with POAG or NTG alone [18]– this screening effort yielded four sequence variations. On screening of the gene in 200 POAG patients of eastern India, we detected two nucleotide variants with potential to be causal to the pathogenesis for the disease, a rare variant and a SNP. The two novel, missense variants were absent in 100 controls suggesting potential role of the variant alleles in POAG. While our study suggests involvement of *OPTC *in causation of POAG, we could not directly implicate the gene for lack of current knowledge on its biological function.

A translationally silent variation (c 602C>T, p.Phe162Phe) present in *OPTC *of a POAG patient but none of the controls suggested potential biological relevance of apparently innocuous change. Though *in silico* analysis does not suggest alteration of splice site resulting from the nucleotide change, wet lab experiments including construction of minigene and its analysis by transcription is necessary for a final answer. Although, mRNA secondary structure prediction analysis did not show a large alteration of stability as measured by change in the structure energy, the structural alteration appeared to be impressive. But a clear observation based on the loop structure and its stability is not apparent to offer further explanation. However, the influence of RNA secondary structure on gene expression and pattern of interaction has been already reported in prokaryotic system [26] and HIV [27,28]. Therefore, it is possible that the lower translational efficiency of the mutant allele might be due to lower stability of the mutant mRNA or, in a less likely situation, structural hindrance in translation. The QRT data demonstrates lower amount of mutant mRNA available for translation. The variation in the stability of the wildtype and mutant mRNA could be measured by limited RNAse digestion or in vitro transcription techniques. It is worthwhile to mention here that it remains important to show functional correlation of the suspected opticin variants with glaucoma pathogenesis to further substantiate role of the gene in the disease process.

A direct correlation of the *OPTC *silent change, described in this article, with POAG would require demonstration of either cosegregation of the variant allele with POAG in a family or identification of the nucleotide change in multiple unrelated patients. Presence of the 'functional silent variation' (c.602 C>T) in the POAG 'suspect' younger sib of the patient, however, supports further of its potential association with POAG.

## Conclusion

We detected two novel missense alterations along with a 'silent' change in *OPTC*; among these p.Glu66Gly and the silent change (p.Phe162Phe) are likely to be associated with glaucomatous phenotype. The expression of the protein from the rare allele (c.602T) of the 'silent' change (c.602C>T) was significantly lower. Therefore, our study highlights the importance of investigating the 'silent' mutations for functional implication that might not be apparent from only *in silico* analysis. Additional studies on role of *OPTC *in POAG would provide better understanding of the function of the gene in the POAG related pathogenesis.

## Methods

### Selection of subjects and collection of blood samples

Two hundred unrelated POAG patients, comprising 156 sporadic cases and 44 with family history, presenting at the Regional Institute of Ophthalmology and Dristi Pradip Kolkata, India, were enrolled in this study. The inclusion criteria involved significant cupping of the optic disc with or without peripapillary changes and presence of open angles on gonioscopy. Cupping >0.6 raised suspicion of glaucoma. If cupping was associated with inferior notching of rim of disc, it was considered to be highly suspicious of being glaucomatous. Also, large cup in a small disc raises suspicion of glaucoma. However, often cup with large disc may be considered as physiological cupping and hence normal. Therefore, it was always confirmed by typical reproducible visual field changes and investigating retinal nerve fiber configuration through direct evaluation of retinal nerve fiber layer (RNFL). Subjects meeting the above criteria with elevated IOP were enrolled as having high tension glaucoma (HTG), and those without elevated IOP were enrolled as patients with normal tension glaucoma (NTG). Patients with history of inflammation or ocular trauma and other systemic conditions were excluded. Based on these criteria, among the patients 34 had juvenile onset open-angle glaucoma (< 30 years of age) and 166 had adult-onset open-angle glaucoma including 87 NTG cases. The mean age of diagnosis of the patients was 52.43 ± 19.33 years with a range varying between 7 years and 84 years. Diagnosis of POAG was confirmed by three of us (SKDT, AKB and AS). Ocular examinations involved measurement of intra ocular pressure (IOP) by Applanation tonometer (Goldmann). Gonioscopy by Goldmann 3-mirror gonioscope (Shaffer's grading), optic disc evaluation (+78D lens), fundoscopy and visual fields testing (Humphrey's automated perimeter).

One hundred ethnically matched normal volunteers from the same ethnic and geographical background of the patients without any history of ocular or systemic diseases served as controls. The controls were examined by direct ophthalmoscopy, thorough examination of optic disc, intraocular tension, gonioscopy, automated visual field analysis and RNFL analysis with the help of scanning laser polarimetry (SLP) and did not have any signs or symptoms of glaucoma The mean age of the controls was 53.86 ± 8.92 years varying between 40 years and 80 years. The patient and control samples were age matched (t = 0.877, df = 298, p = 0.3812).

Around 10.0 ml of peripheral blood was collected from the patients and normal volunteers by venipuncture with informed consent. Genomic DNA was prepared from whole blood using the conventional phenol-chloroform method [29]. The study protocol adhered to the tenets of the Declaration of Helsinki and was approved by the Institutional Review Board.

### Polymerase chain reaction (PCR)

PCR was carried out in a total volume of 25.0 μl containing 50–100 ng genomic DNA, 0.4 μM of each primer, 0.2 mM of each dNTP, appropriate concentration of MgCl_2_, and 0.5 unit of *Taq polymerase *(Invitrogen, Carlsbad, CA) in a thermocycler (GeneAmp-9700, PE Applied Biosystems). All the coding exons along with the intron-exon boundaries were amplified using appropriate primers (Table 2).

### *OPTC *screening by single stranded conformational polymorphism (SSCP) and denaturing high-performance liquid chromatography (DHPLC)

The PCR products were run in the pre-cast (12.5% or 15%) SSCP gels. The experimental conditions were adjusted as per the recommendations of the manufacturer (Amersham, Piscataway, USA). All the patient samples (n = 200) were screened for allelic variation by SSCP. PCR products showing mobility shift were sequenced for characterizing the variation. Among the four nucleotide variants identified, two (c.313A>G, p.Glu66Gly & c.382 T>C, p.Ile89Thr) were screened in controls (n = 100) by dHPLC (The WAVE system, Transgenomic Inc., Omaha, USA) under partially denaturing conditions. Prior to loading, the amplicons were subjected to heteroduplex formation and subsequently run under melting temperatures calculated from the WAVEMAKER™ software (version 4.1). The DNA samples containing the variant alleles were used as positive controls in the experiment. Variants were observed as heteroduplex peaks.

### DNA sequencing and bioinformatic analysis

The amplicons were column-purified using Qiagen PCR-purification kit (Qiagen, Hilden, Germany), and the patient samples showing mobility shift in SSCP were further characterized by sequencing using the BigDye termination chemistry (version 3.1) in an ABI 3130XL capillary DNA sequencer (Applied Biosystems, Foster City, CA) following manufacturer's protocol. The data was analysed using SEQUENCING ANALYSIS software (version 5.2).

The observed nucleotide sequences in the patients were compared to the *OPTC *wild type sequence [GenBank: NM_014359] for variations using Pair wise BLAST [30]. All novel mutations detected in *OPTC *were checked for their functional relevance by the SIFT (Sorting Intolerant From Tolerant)[31] homology tool, using related sequences of plants and animals from a BLAST search of GenBank to predict whether a substitution was likely to have any phenotypic effect (Table 1). It essentially predicts the potential of a substituted amino acid to be deleterious in a protein sequence. A score less than 0.05 is the benchmark of deleterious change [32]. Generations of any cryptic splice site for any nucleotide variation was checked using ESE finder (exon splicing enhancer finder) [33] or RESQUE-ESE (relative enhancer and silencer classification by unanimous enrichment) [34]. ESEfinder is a predictive analysis derived from functional *in vivo *and *in vitro *systematic evolution of ligands by exponential enrichment (SELEX) and statistical analysis [35]. RESCUE-ESE predicts which sequences have ESE activity by statistical analysis of exon-intron and splice site junction [36]. RNADraw [37] was used to predict the deviation from the normal RNA secondary structure for the synonymous variation (p.Phe162Phe).

### Screening of control samples

Among the four nucleotide variants identified (c.919T>C, p.Leu268Pro) only one caused change in a restriction site (Bpu10 I). Therefore, this variant was screened in controls by an RFLP assay using the restriction enzyme (New England BioLabs, Boston, MA). The silent change (c.602C>T, p.Phe162Phe) was screened by direct sequencing to make sure that apparently innocuous variant was not present in any control sample. The other two variants (c.313A>G, p.Glu66Gly & c.382 T>C, p.Ile89Thr) which could be easily discriminated from wild type alleles by dHPLC was screened in the controls by this method for convenience and speed.

### Site directed mutagenesis

Opticin constructs in pEGFPN1 mammalian expression vector (kindly gifted by Dr. Michael Walter, Dept. of Medical Genetics, University of Alberta) were initially isolated from a dam^+ ^strain. The oligonucleotide used for changing C to T at 602nd nucleotide in *OPTC *cDNA was 5'- GCC TAC CTG TAT GCA CGC TTT AAC CGC ATC AGC CGT ATC AG -3' and 5'- CTG ATA CGG CTG ATG CGG TTA AAG CGT GCA TAC AGG TAG GC -3'. Site-directed mutagenesis was performed using the QuikChange XL Site-Directed Mutagenesis Kit (Stratagene, La Jolla, CA, USA) according to the manufacturer's protocol. After the reaction was over, the residual product contained only the nicked mutated plasmid without any starting material, which was then transformed into *XL10-GOLD ultra competent *cells (A derivative of XL2 Blue MRF', Stratagene, La Jolla, CA, USA) supplied by Stratagene. The transformation reaction in E. coli *XL10-GOLD* was carried out as per the protocol provided by the manufacturer (Stratagene, La Jolla, CA, USA). Plasmid isolation was done for both c.602C and c.602T variant cloned in pEGFPN1 vector using QIAGEN plasmid midi kit following the protocol provided by manufacturers (Qiagen, Germany). The selection of the mutant clone as designed was confirmed by sequencing the inserts within the mutagenized recombinant clone.

### Mammalian cell culture

The human retinal pigment epithelium cell line RPE8319 (kind gift from Dr. Frans Cremers, University Medical Center, Nijmegen, The Netherlands) was maintained in DMEM (Dulbecco's modified Eagle Medium, GIBCO BRL) at pH 7.4 supplemented with 10% fetal bovine serum (GIBCO BRL) containing penicillin/streptomycin/gentamycin in the presence of 5% CO_2 _at 37°C. Transient transfection of human RPE cell lines was performed with the Lipofectamine 2000 system according to the manufacturer's instructions (Invitrogen, Carlsbad, CA). Cells were harvested after 40 hours of transfection, both for RNA extraction for QRTand protein lysate preparation for western blot.

### RNA extraction and cDNA preparation

Total RNA was isolated from the transfected or untransfected RPE cells using TRIZOL (Invitrogen, Carlsbad, CA). Two micrograms of total RNA was reverse-transcribed using first strand cDNA synthesis kit (Invitrogen, Carlsbad, CA), in a total volume of 20 μl and stored at -20°C until use.

### Quantitative RT-PCR for *OPTC *c.602C and c.602T variant expression

The mRNA expression of normal (c.602C) and mutant (c.602T) variant of *OPTC *was determined by real-time RT-PCR in the 7500 Real Time PCR System (Applied Biosystems, Foster City, CA) using SYBR Green Jumpstart Taq Ready Mix (Sigma, St.Louis, MO). The following primers were used: OPTC TF 5' CTCTGCCCGTGCTGCCCAGT 3', OPTC TR 5' GGCAAAGGCCCCGGGATAGA 3' and human β-actin F 5'-TGACGGGGTCACCCACACTGTGCCCATCTA-3', human β-actin R 5'-CTAGAAGCATTGCGGTGGACGATGGAGGG-3'. The threshold cycle C_T _of duplicate samples was determined using 7500 System SDS software (Applied Biosystems, Foster City, CA). The levels of both variants (c.602C & c.602T) of *OPTC *were normalized to β-actin levels by calculating the ΔC_T_value, which is the C_T _(threshold cycle) of the housekeeping gene (β-actin) subtracted from the C_T _of the target gene (*OPTC*). As OPTC mRNA is reportedly expressed in RPE cells, we further normalize the data by calculating the ΔΔC_T _value which is a subtraction of ΔC_T_value of untransfected cells from each ΔC_T _value of both cells transfected with c.602C & c.602T variant of *OPTC*. The mean ΔΔC_T _value of each group (c.602C & C.602T) was represented in corresponding figure (Fig 4A). All calculations were done according to the published paper in 2001 by Giulietti et al. [38].

### Western Blot analysis

For western blot analysis transfected or untransfected RPE cells were harvested in TBST [50 mM Tris-HCl (pH7.5), 15 mM EDTA, 150 mM NaCl, 0.1% TritonX-100], the lysis buffer, supplemented with protease inhibitor cocktail (1 ul/10^6 ^cell; Sigma, St.Louis, MO) by freeze-fracture. Cell extracts were then subjected to Bradford assay for total protein estimation that would be necessary for equal loading of cell lysate. The whole cell lysates were resolved in 10–12% SDS-PAGE in constant current and the gel was transferred to PVDF membrane (Amersham Inc, The Netherlands). After transfer the membrane was stained with Ponceau-S solution and the gel with Coomassie blue to ensure efficient transfer of the proteins to the membrane. The membrane was blocked with 5% BSA in TBST for at least 2 hrs at room temperature followed by addition of human specific opticin antibody (kind gift from Dr. Paul Bishop, University of Manchester, UK) and beta-actin (Sigma, St. Louis, MO) antibody (1:500–1000 dilution), raised in rabbit and mouse respectively, for overnight reaction at 4–8°C. Then the membrane was washed with TBST (0.05% of tween-20 in TBS) thrice for 10–15 mins each time. After that suitable secondary antibody (goat anti mouse or anti rabbit) conjugated with ALP or HRP (1:1000 dilution) was added and incubated for 3 hrs at room temperature. Next, the membrane was washed again with TBST 6 times for 10 mins each. In case of the ALP fused secondary antibodies the membrane was treated with BCIP/NBT (Genei, India) at pH 8.8 for 15–20 mins. For HRP fused secondary antibodies the membrane was treated with TMB/H_2_O_2 _(Genei, India) for 10–15 mins. Colored bands were observed in the membrane due to chromogenic reaction.

## Authors' contributions

MA carried out the *in silico*, functional and molecular genetic studies, and drafted the manuscript. SM and AB carried out the molecular genetic studies. SKDT, AKB & AS selected the patients and controls and made substantial contribution in acquisition, analysis and interpretation of clinical data. SC carried out the DHPLC analysis and involved in drafting the manuscript for important intellectual content. KR conceived the study, led the group in designing experimental strategies and provided intellectual input for giving final shape of the manuscript. All authors read and approved the final manuscript.

## Figures and Tables

**Table 1:** Clinical data of POAG patients having sequence changes in *OPTC*

**Patient ID (age at diagnosis)**	**Location**	**Mutation **	**SIFT score**^a^	**Glaucoma subtype**	**Visual acuity RE, LE**	**IOP (mm of Hg) RE, LE**	**C:D ratio RE, LE**	**Visual field changes**
GL131 (37 yrs)	Exon 2	Glu66Gly*	0.03	JOAG	No PL, FC(1ft) & PL+ PR	16^b^, 42	media hazy, 0.8	Glaucomatous field changes
GL77 (65 yrs)	Exon 3	Ile89Thr*	0.66	POAG	6/12, 6/6	25,18	0.8, 0.7	Glaucomatous field changes
GL42 (32 yrs)	Exon 4	Phe162Phe*	NA	JOAG	6/18, NoPL	17, ND	0.8, ND	Localized defect near fixation point in the infero-temporal quadrant and tubular vision in right eye

*Novel variant; ^**a**^SIFT score predicts phenotypic effect; < 0.05 predicted to be deleterious, =/>0.05 tolerated [31]; ^b^The IOP in right eye of GL131 is post-operative value.

The cDNA numbers correspond to Genbank accession number NM_014359

**Abbreviations used: **RE: right eye and LE: left eye; IOP: Intra ocular pressure; C:D ratio: cup to disc ratio; PO: post operative; BE: both eye; PL: perception of light; PR: projection of rays; FC: finger counting; ND: not detectable due to Band keratopathy in left eye

**Table 2:** Primer sequences and PCR conditions for amplifying coding sequence of *OPTC*

**Primer Sequence**		**Location**	**Anneal temp. (°C)**	**Mg**^2+^**conc. (mM)**	**Length of PCR product (bp)**
				
**Forward (5'-3')**	**Reverse (5'-3')**				
2aF-GTGGAGTAAATCTGCGAGCC	2aR-GGCAGAACTTCAAAGGAATC	Exon 2	50	1.5	264
2bF-CTGAAGGGATTCTCAGTCCCATCTG	2bR-CTCCCAGTGTCATGCAGGGAATGT	Exon 2	62	2.0	313
3F-GTTCCCAGAGTCCAAAGTTAAG	3R-GGTCATAGCTGCTTGGCCTATG	Exon 3	54	1.5	275
4F-CAGATGGCCATGGTTCAGACAC	4R-GTGTTGGTGACTGTCCTAGTG	Exon 4	58	2.0	333
5F-GACAAGGAAAGATAGTGTGTTCTG	5R-GTGGGTGGTGGAGGTGATAGATAG	Exon 5	60	2.0	310
6F-CCACCTGTCTCTGGTCTCATAG	6R-CATCCTCCACGCTGTTTATCACC	Exon 6	56	1.5	233
7F-CTGATGTGAGCCTTTGGTGCTGC	7R-GATAAGGAATTGGGCTTTATGGC	Exon 7	62	2.0	334
